# Effects of Traditional Chinese Medicinal Plants on Anti-insulin Resistance Bioactivity of DXMS-Induced Insulin Resistant HepG2 Cells

**DOI:** 10.1007/s13659-014-0028-0

**Published:** 2014-07-10

**Authors:** Jun-Zeng Ma, Li-Xin Yang, Xiao-Ling Shen, Ji-Huan Qin, Li-Lan Deng, Selena Ahmed, Hong-Xi Xu, Da-Yuan Xue, Jiang-Xia Ye, Gang Xu

**Affiliations:** 1 State Key Laboratory of Phytochemistry and Plant Resources in West China, Kunming Institute of Botany, Chinese Academy of Sciences, Kunming, 650201 People’s Republic of China; 2 College of Life and Environmental Science, Minzu University of China, 27 ZhongGuanCun South Avenue, Beijing, 100086 China; 3 Laboratory of Chinese Herbal Drug Discovery, Tropical Medicine Institute, Guangzhou University of Chinese Medicine, Guangzhou, 510405 People’s Republic of China; 4 Southwest Forestry University, Kunming, 650224 People’s Republic of China; 5 Sustainable Food and Bioenergy Systems Program, Department of Health and Human Development, Montana State University, Bozeman, MT 59717 USA; 6 Shanghai University of Traditional Chinese Medicine, Shanghai, 201203 People’s Republic of China

**Keywords:** Traditional medicinal plants, Diabetes, Anti-insulin resistance bioactivity, DXMS-induced insulin resistant HepG2 cells

## Abstract

Medicinal plants have a long history of use in China to treat diabetic symptoms. Ancient Chinese medical manuscripts and ethnobotanical surveys document plant remedies that continue to be actively used in China for the treatment of diabetic symptoms. Based on a systematic ancient Chinese medical manuscripts review in combination with ethnobotanical survey, 16 medicinal plants for the traditional treatment of diabetic symptoms were identified for the evaluation of anti-insulin resistance bioactivity. The biological activity of 16 medicinal plants was tested on dexamethasone (DXMS)-induced insulin resistant HepG2 cells. The result shows that 11 of the 16 medicinal plants enhanced glucose uptake of DXMS-induced insulin resistant HepG2 cells, thereby demonstrating their ability to increase insulin sensitivity, other five medicinal plants including *Astragalus membranaceus* were found ineffective. The study shows that ancient Chinese medical manuscripts and ethnobotanical surveys on plants for the prevention and treatment of diabetic symptoms provide a promising knowledge base for drug discovery to mitigate the global diabetes epidemic.

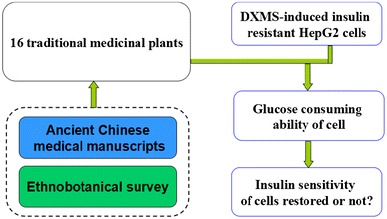

## Introduction

Diabetes mellitus (DM) is an increasingly prevalent group of metabolic diseases affecting hundreds of millions of people worldwide and costing billions of dollars in healthcare. This heterogeneous group of disorders is characterized by hyperglycemia [1] and results from absolute insulin deficiency, insulin resistance and/or abnormal insulin secretion [2]. DM may lead to a series of complications including blindness, renal failure, nerve damage, stroke, and limb amputation [3]. In 2012, there were approximately 4.8 million deaths resulting from DM with 471 billion USD spent on healthcare costs globally. Approximately 371 million people today have DM [4].

Currently available drugs for the treatment of DM include sulphonylureas, biguanides, *α*-glucosidase inhibitors, and thiazolidinediones [5]. However, these treatments are noted to have varying degrees of adverse side effects such as gastrointestinal disturbances and hypoglycemia [6]. Given the side effects from current treatments, it is necessary to search safer agents for the treatment of DM, including remedies that may be more efficacious. Traditional medicinal plants have an extensive history for the treatment of diseases [7] and offer promising leads for the treatment of DM. Increased attention has been given to these resources for their complementary therapeutic effects to supplement western medicine [8]. Ethnopharmacological studies have noted that many traditional remedies have relatively fewer side-effects, better patient tolerance in diverse cultural contexts, less toxicity, and lower costs compared to modern drugs for DM treatment [9].

Traditional Chinese Medicine (TCM) is a major global healthcare system that relies on medicinal plants and plays a crucial role in healthcare for hundreds of millions of people in China including for the treatment of DM. In TCM, DM is categorized as “xiaoke” (polydispsia) with complex symptoms of excessive eating, drinking, polyuria, emaciation and urine-sweeting [10].

“Xiaoke” is recorded in many ancient Chinese medical manuscripts including “Shen Nong Ben Cao Jing”, “Ming Yi Bie Lu”, “Bei Ji Qian Jin Yao Fang”, “Qian Jin Yi Fang”, “Dian Nan Ben Cao”, and “Ben Cao Gang Mu”. Numerous medicinal plants have been reported in TCM for the treatment of “xiaoke” [9, 11]. For example, a powdered mixture of *Coptis chinensis* Franch. (Ranunculaceae), *Astragalus membranaceus* var. *mongholicus* (Bunge) P.K. Hsiao. (Leguminosae) and *Lonicera japonic* Thunb. (Caprifoliaceae) was found to improve insulin resistance for Type 2 DM [11]. Several medicinal plants from traditional Chinese pharmacopeia have been found to exert beneficial action on diabetes and related complications via multi-mechanisms [9].

The rising interest and use of traditional plant remedies has been met with concerns over the lack of quality control and scientific evidence for the efficacy and safety of herbal medicine [12]. Scientific research has responded to this concern with increased attempts to search for common ground between traditional healing systems and western medicine including clinical and animal studies to validate the efficacy and safety of traditional herbal medicine [13, 14]. However, few studies have been devoted to investigating the anti-diabetic activity of traditional medicinal plants by integrating ethnobotanical, phytochemical, and pharmacological approaches. The objective of this study is to evaluate the anti-insulin resistance bioactivity of 16 selected traditional Chinese medicinal plants in DXMS-induced IR (insulin resistant) HepG2 cells. HepG2 cells are a pure cell line of human liver carcinoma derived from the liver tissue with a well-differentiated hepatocellular carcinoma [15]. The model of DXMS-induced insulin resistance in HepG2 cells is recognized as important for interpreting insulin resistance mechanisms and drug screening [16].

## Results and Discussion

### Inventory of Target Anti-diabetic Plants

Table 1 shows traditional medicinal plants used in the treatment of diabetic symptoms. Plants of Group I were found to be mentioned with relatively high frequency in the literature review for their anti-diabetic activities including: *A. membranaceus*, *C. chinensis*, *Morus alba*, *Pueraria lobata*, *Trichosanthes kirilowii*, *Alisma orientale*, *Scrophularia ningpoensis*, *Cuscuta chinensis*, *Schisandra chinensis*. Of these, seven plants were mentioned with high frequency for the treatment of “xiaoke” and diabetes reported in articles published between 1980 and 2003 including: *A. membranaceus*, *C. chinensis*, *P. lobata*, *T. kirilowii*, *A. orientale*, *S. ningpoensis* and *S. chinensis* [17–20]. Plants of Group II were collected through ethnobotanical survey in Lijiang, Dali, and Dongchuan etc. during 2011–2013. Plants were identified through semi-structured interviews with traditional herb doctors. Medicinal plants usage frequency of Han, Naxi, Bai and Lisu socio-linguistic groups for the treatment of “xiaoke” and diabetic symptoms was studied.

**Table 1:** List of traditional medicinal plants used in the treatment of diabetic symptoms

No.	Latin name	Family	Local name	Used part	Function	Frequency^a^	Source^b^	Yield (%) MEs	Voucher
A1	*Astragalus membranaceus* var. *mongholicus* (Bunge) P.K. Hsiao.	Leguminosae	Huang Qi	Root	Tonifying Qi and lifting yang, inducing diuresis for removing edema	50	7	22.8	KUNX01
A2	*Coptis chinensis* Franch.	Ranunculaceae	Huang Lian	Root	“Xiaoke” and excessive urine, urine like oil	69	2, 3, 4, 5, 6, 7	19.0	KUNX02
A3	*Morus alba* Linn.	Moraceae	Sang Bai Pi	Root skin	Inducing urination, to treat “xiaoke” and excessive urine	3	4, 6	11.6	KUNX03
A4	*Pueraria lobata* (Willd.) Ohwi.	Leguminosae	Ge Gen	Root	To treat “xiaoke”, heat, stomach weaken, dysphoria	57	1, 2, 3, 4, 5, 7	6.7	KUNX04
A5	*Trichosanthes kirilowii* Maxim.	Cucurbitaceae	Tian Hua Fen	Root	To treat “xiaoke”, dysphoria, and heat	66	1, 2, 3, 4, 6, 7	3.9	KUNX05
A6	*Alisma orientale* (Samuel.) Juz.	Alismataceae	Ze Xie	Root	To treat “xiaoke”	58	2, 3, 4, 7	12.6	KUNX07
A7	*Scrophularia ningpoensis* Hemsl.	Scrophulariaceae	Xuan Shen	Root	To treat polydipsia and pyreticosis, removing heat to cool blood	50	7	22.4	KUNX08
A8	*Cuscuta chinensis* Lam.	Convolvulaceae	Tu Si Zi	Seeds	To treat “xiaoke” and dribbling urination	3	3, 4, 6	11.5	KUNX09
A9	*Schisandra chinensis* (Turcz) Baill. Hist. PI.	Magnoliaceae	Wu Wei Zi	Fruits	To treat edema from nephritis, using diuretic of hydragogue to alleviate water retention	53	3, 4, 7	43.9	KUNX10
A10	*Viburnum odoratissimum* Ker-Gawl.	Caprifoliaceae	Jia Mi	Branch leaves	Removing heat to cool blood, inducing diuresis to alleviate edema, diffusing wind-heat, clearing heat-fire, tonifying spleen and dampness	12	Dali	13.2	KUNX11
A11	*Polygonatum verticillatum* (L.) All.	Liliaceae	Lun Ye Huang Jing	Bulb	Tonifying spleen and dampness, “xiaoke”, tonifying Qi	8	Lijiang	9.7	KUNX12
A12	*Hypericum henryi* Levl. et fan.	Clusiaceae	Xi Nan Jin Si Mei	Aerial plant	Clearing away heat and toxic materials, diuresis, promoting blood circulation to restore menstrual flow	3	Redland	10.5	KUNX14
A13	*Lobaria yunnanensis* Yoshim.	Lobariaceae	Qing Wa Pi	Whole plant	Inducing diuresis to alleviate edema	7	Dali	7.3	KUNX17
A14	*Agrimonia pilosa* Ldb.	Rosaceae	Xian He Cao	Whole plant	Promoting blood circulation to restore menstrual flow	9	Lijiang	9.1	KUNX18
A15	*Fragaria nilgerrensis* Schlecht. ex Gay var*. mairei* (Levl.) Hand.-Mazz.	Rosaceae	Ye Cao Mei	Whole plant	Clearing heat	6	Lijiang	16.6	KUNX19
A16	*Fagopyrum dibotrys* (D. Don) *Hara*	Polygonaceae	Jin Qiao Mai	Root	Tonifying spleen and dampness	10	Lijiang	20.2	KUNX20

^a^Frequency means recorded frequency in ancient Chinese medical manuscripts or experiences from herbalists for diabetes treatment

^b^1 “Shen Nong Ben Cao Jing”, 2 “Ming Yi Bie Lu”, 3 “Bei Ji Qian Jin Yao Fang”, 4 “Qian Jin Yi Fang”, 5 “Dian Nan Ben Cao”, and 6 “Ben Cao Gang Mu”, 7 Ref. [21]

Table 2 shows review of previous studies of traditional Chinese medicinal plants for treating diabetic symptoms including: *A. membranaceus*, *C. chinensis*, *M. alba*, *P. lobata*, *T. kirilowii*, *Agrimonia pilosa*, *C. chinensis*, *S. chinensis* and *A. orientale*. These medicinal plants can be categorized according to four types of activities for treating DM including: (1) improving glucose metabolism, (2) enhancing insulin sensitivity or insulin levels, (3) regulating lipid metabolism, (4) improving pancreatic function. Table 2 also lists the anti-diabetic constituents of plants identified through a literature review for the treatment of DM symptoms as well as the mechanisms involved in their reported bioactivity.

**Table 2:** Review of previous studies of traditional Chinese medicinal plants for treating diabetic symptoms

No.	Latin name	Anti-diabetic constituents reported	Activity*	Mechanism	References
A1	*Astragalus membranaceus*	Isoastragaloside I, Astragaloside II and IV	b	Elevating adiponectin production	[25, 26]
		Formononetin, calycosin	c	Activating PPAR*α* and PPAR*γ*	[27]
		Astragalus polysaccharide	b	Regulating PKB/GLUT4 signaling in skeletal muscle, inhibiting PTP1B	[28, 29]
A2	*Coptis chinensis*	Berberine	b	Modulating the structure of gut microbiota	[30]
			a	Stimulating AMPK*α*1, AMPK*α*2 and inhibiting hepatic gluconeogenesis	[31, 32]
		Polysaccharide	a, b		[33]
A3	*Morus alba*	Polysaccharides	a, d	Inhibiting inflammatory response and attenuate oxidative stress in pancreas tissue	[34]
		Moracin M, Mullberroside A etc.	a		[35]
		Extract	a, b		[36]
A4	*Pueraria lobata*	Puerarin	a, c, d	Promoting expression of insulin etc., activating *α* 1-adrenoceptors. Upregulating the expression of PPAR*γ*	[37–40]
A5	*Trichosanthes kirilowii*	Lectin	a	Increasing glucose uptake of liver cells	[41]
A6	*Alisma orientale*		a	Inhibiting *α*-glucosidase activity	[42, 43]
A8	*Cuscuta chinensis*	Polysaccharide	a, c	Inhibiting *α*-amylase activity	[44, 45]
A9	*Schisandra chinensis*	Lignan-rich fraction	a, b	Activating PPAR-*γ*	[46]
A14	*Agrimonia pilosa*	1*β*-Hydroxy-2-oxopomolic acid	c	Blocking PPAR*γ* and C/EBP*α* expression	[47]

* a, improving glucose metabolism; b, improving insulin sensitivity or enhancing insulin level; c, regulating lipid metabolism; d, improving the function of pancreas

Seven plants have not been previously evaluated for their anti-diabetic activity including: *Hypericum henryi*, *S. ningpoensis*, *Viburnum odoratissimum*, *Lobaria yunnanensis*, *Fragaria nilgerrensis*, *Polygonatum verticillatum* and *Fagopyrum dibotrys.* Although no anti-diabetic research has been reported on *H. henryi*, extracts of other species in the genus of *Hypericum* including *Hypericum ascyron* [21] and *Hypericum perforatum* [22] have shown anti-diabetes related activity. Similarly, while *P. verticillatum* and *F. dibotrys* have not previously been reported for diabetic related activity, the extract of other species in the genera *Polygonatum* and *Fagopyrum* have been found to show hypoglycemic effects including *Polygonatum Sibiricum* [23] and *Fagopyrum tataricum* [24].

### Assay Results of Traditional Medicinal Plants

According to GCA_IR(Extract)_ (Glucose Consuming Ability) values, 11 of the 16 extracts were effective in increasing anti-insulin resistance bioactivity of DXMS-induced IR HepG2 cells at three test concentrations. Table 3 lists Relative Glucose Consumption, Cell Viability and Glucose Consumption Ability of medicinal plants. HepG2 cells treated with 1 μmol/L of DXMS (IR control) exhibited comparable cell viabilities (CV_IR_ > 95 %) to DXMS free insulin sensitive HepG2 cells (IS control) but consumed much less glucose (RGC_IR_ = 82.1 %, RGC: Relative Glucose Consumption), implying that insulin resistance in HepG2 cells was successfully induced by DXMS. The IR state of cells was further supported by a GCA_IR_ value of 0.86.

**Table 3:** Anti-insulin resistance bioactivity of 16 extracts on DXMS-induced IR HepG2 cells

Sample	C (μg/mL)	RGC (%)	CV (%)	GCA	Comments
*Alisma orientale*	100	105.0 ± 4.4*	103.2 ± 5.1	1.02	Highly effective and non-toxic Highly potential
	50	97.2 ± 7.2	104.5 ± 2.9	0.93	
	25	84.2 ± 2.1	100.7 ± 0.1	0.83	
*Viburnum odoratissimum*	100	88.6 ± 4.8	68.8 ± 8.9	1.29	Highly effective at toxic concentrations
	50	88.0 ± 5.6	85.2 ± 3.0	1.03	
	25	89.6 ± 9.5	92.6 ± 1.8	0.97	
*Coptis chinensis*	100	75.9 ± 3.5	23.3 ± 1.9	3.26	Highly effective but toxic
	50	94.4 ± 8.8	43.0 ± 5.4	2.19	
	25	142.4 ± 0.1	65.6 ± 5.8	2.17	
*Morus alba*	100	82.6 ± 2.0	45.6 ± 2.1	1.81	Highly effective but toxic
	50	90.1 ± 5.1	65.9 ± 8.0	1.37	
	25	83.9 ± 1.7	80.2 ± 14.3	1.05	
*Hypericum henryi*	100	82.9 ± 0.3	51.0 ± 4.9	1.62	Highly effective but toxic
	50	110.8 ± 1.0**	71.8 ± 6.6	1.54	
	25	94.6 ± 1.6*	77.7 ± 1.3	1.22	
*Fragaria nilgerrensis*	100	100.0 ± 3.3**	87.9 ± 4.4	1.14	Highly effective but toxic
	50	96.8 ± 2.6**	88.2 ± 5.1	1.10	
	25	95.7 ± 6.9*	91.5 ± 7.9	1.04	
*Fagopyrum dibotrys*	100	37.6 ± 0.6	4.7 ± 2.4	8.00	Highly effective but toxic
	50	47.5 ± 2.1	20.5 ± 2.8	2.32	
	25	71.1 ± 3.4	69.8 ± 6.3	1.02	
*Schisandra chinensis*	100	71.6 ± 2.6	79.6 ± 3.8	0.90	Moderately effective
	50	86.8 ± 5.0	96.3 ± 3.4	0.90	
	25	96.6 ± 4.9	101.3 ± 2.7	0.95	
*Pueraria lobata*	100	85.8 ± 8.4	87.7 ± 7.4	0.98	Moderately effective with low toxicity
	50	85.4 ± 6.6	90.9 ± 8.6	0.94	
	25	83.5 ± 4.5	89.2 ± 6.3	0.94	
*Trichosanthes kirilowii*	100	75.5 ± 5.9	85.8 ± 2.6	0.88	Moderately effective with low toxicity
	50	77.4 ± 6.4	83.6 ± 4.3	0.93	
	25	81.6 ± 8.2	87.8 ± 4.8	0.93	
*Lobaria yunnanensis*	100	82.5 ± 5.3	85.6 ± 8.1	0.96	Moderately effective with low toxicity
	50	80.8 ± 6.4	90.0 ± 7.0	0.90	
	25	68.2 ± 6.2	89.7 ± 6.8	0.76	
*Astragalus membranaceus*	100	68.8 ± 1.6	89.0 ± 10.3	0.77	Ineffective
	50	71.3 ± 1.1	88.6 ± 7.5	0.80	
	25	68.8 ± 1. 4	92.1 ± 6.2	0.75	
*Scrophularia ningpoensis*	100	71.5 ± 6.7	85.8 ± 6.5	0.83	Ineffective
	50	65.7 ± 7.9	89.0 ± 5.7	0.74	
	25	66.2 ± 6.2	90.2 ± 4.8	0.73	
*Cuscuta chinensis*	100	78.5 ± 3.9	91.3 ± 9.2	0.86	Ineffective
	50	80.3 ± 4.9	93.2 ± 9.8	0.86	
	25	78.8 ± 4.4	101.0 ± 3.8	0.78	
*Polygonatum verticillatum*	100	70.6 ± 1.6	87.8 ± 7.8	0.80	Ineffective
	50	73.8 ± 0.2	86.5 ± 4.6	0.85	
	25	75.3 ± 7.5	87.0 ± 3.0	0.86	
*Agrimonia pilosa*	100	56.7 ± 3.7	85.2 ± 3.9	0.66	Ineffective
	50	59.7 ± 1.5	90.7 ± 6.8	0.66	
	25	69.1 ± 2.5	92.8 ± 5.8	0.74	
IR control		82.1 ± 5.1^▲^	95.2 ± 3.9	0.86	
IS control		100	100	1.00	

RGC and CV were expressed as Mean value ± standard deviation (n = 3)

GCA ≥ 1, highly effective; 0.86 < GCA < 1, moderately effective; GCA < 0.86, ineffective

Compared to IS control, data were significantly different at ^▲^*P* < 0.05

Compared to IR control, data were significantly different at * *P* < 0.05, ** *P* < 0.01 and *** *P* < 0.001

Among of the 16 analyzed medicinal plants, *A. orientale* was found to be both highly effective as well as no toxicity. Five species including *C. chinensis*, *M. alba*, *F. dibotrys*, *H. henryi* and *F. nilgerrensis* were shown to be highly effective but toxic. One species, *V. odoratissimum*, was found to be highly effective but only at toxic concentrations. *S. chinensis* was found to be moderately effective. Three species, *P. lobata*, *T. kirilowii*, *L. yunnanensis*, were shown to be moderately effective with low toxicity. The remaining five of the 16 test extracts from *A. membranaceus*, *S. ningpoensis*, *C. chinensis*, *P. verticillatum*, *Agrimonia pilosa* were found to be ineffective as anti-insulin resistance agents in DXMS-induced IR HepG2 cells.

### Anti-insulin Resistance Bioactivity of Traditional Medicinal Plants

This study supports that traditional Chinese medicinal plants have the potential for development as anti-diabetic drugs. All plants identified through the literature review of traditional Chinese medical texts and through ethnobotanical surveys exhibited efficacy as anti-insulin resistance agents. Eleven of the 16 tested traditional medicinal plants were able to enhance glucose uptake in DXMS-induced IR HepG2 cells, thereby demonstrating their in vitro anti-insulin resistance bioactivity. One of these species, *A. orientale* was found to have both high efficacy and non-toxicity. *A. orientale* is an aquatic herbaceous plant that is cultivated widely in Sichuan, Jiangxi and Fujian Provinces of China and is used as a key ingredient in some TCM prescriptions such as hachimijiogan (Ba Wei Di Huang Wan). Previous studies have shown that hachimijiogan increased insulin secretion and decreased postprandial glucose in type-2 diabetic Goto-Kakizaki rats. In addition, *A. orientale* has shown to have extensive bioactivities including diuresis, modulating immune system, hypotensive and anti-atherosclerosis. Further research is called for towards the development of this plant as a widespread drug for the treatment of DM. In vivo studies are particularly called for as extracts that show efficacy in vitro will not necessarily lead to a corresponding response in vivo due to the dose used and associated metabolic responses.

Six of the nine medicinal plants identified through the literature review exhibited high or moderate anti-insulin resistance bioactivity in this study while five of the seven medicinal plants identified through the ethnobotanical surveys exhibited efficacy in the study screenings. Previous research has analyzed nine of the 16 medicinal plants examined here (Table 2) for their anti-diabetic activity using in vitro and/or in vivo drug screenings and have found these species to be efficacious for improving glucose metabolism through different mechanisms including stimulating insulin secretion of pancreas, increasing insulin sensitivity of IR cells, and delaying intestinal absorption of glucose.

The three plants that have previously been reported to have anti-diabetic activity and had no activity in the present study (*A. membranaceus*, *C. chinensis* and *A. pilosa*) along with the two plants identified in the ethnobotanical surveys that showed no bioactivity (*S. ningpoensis* and *P. verticillatum*) may be due to various factors regarding the specific bioactivity assessed here and the preparation protocols. Anti-insulin resistance bioactivity is one of several indicators of anti-diabetic activity and other mechanisms are involved in diabetes pathology. For example, *β*-hydroxy-2-oxopomolic acid in *Agrimonia pilosa* works through regulating lipogenesis but not through directly affecting the insulin-signaling pathway of cells. Traditional medicinal plants are most often used in complex formulas with other species where each species has a distinct effect and their synergistic effect is responsible for the ultimate bioactivity. The methanol preparation used to evaluate medicinal plants in this study may not best extract active constituents. Methanol is relatively efficient in extracting hydrophobic compounds while water may be more efficient in extracting hydrophilic compounds and macromolecules such as polysaccharides. Previous studies have shown methanol extracts of plant material to exhibit different activities compared to water extracts [48]. Future ethnopharmacological studies on the medicinal plants examined here should incorporate traditional preparation protocols.

### Toxicity of Traditional Medicinal Plants

Ten of the 11 plants that were found to be efficacious in this study demonstrated varying degrees of toxicity to IR HepG2 cells at different concentrations. However, standard methanol laboratory extractions rather than traditional preparations were used for the evaluation of these species. For example, preparation using traditional protocols such as hot water infusions may be less effective in extracting particular hydrophobic compounds that contribute to toxicity. Traditional preparation protocols may also call for more dilute solutions compared to the methanol extractions used in this study. In addition, traditional medicinal remedies are often complex formulas involving multiple plants where one plant may offset the toxicity of another plant. Further research is needed that incorporates traditional preparation of the target medicinal plants found as efficacious towards their potential development as anti-diabetic drugs. In addition, further studies should explore the toxicity of extracts prepared with protocols involving solvents of different polarity and varying concentrations. Further research should also examine toxicity levels in vivo as extracts with toxicity in vitro will not necessarily lead to a corresponding response in vivo due to the dose used and metabolic responses in vivo.

### GCA as an Evaluation Indicator

Findings support that a more effective indicator than the current indicator involving RGC would incorporate cell viability. The glucose consumption assay is commonly used to evaluate the anti-insulin resistance bioactivity of a test sample at non-toxic concentrations. In such cases, increased RGC indicates increased anti-insulin resistance bioactivity of cells by the test sample. In this study, bioactivity of traditional medicinal plants was investigated using their methanol extracts. Crude extracts are complicated in constituents of various chemical structures and diverse bioactivities. Therefore, the active components in test extracts may increase glucose uptake of living cells and at the same time the toxic components in the extract reduce the number of living cells. In such cases, RGC_Extract_ ≤ RGC_Control_ does not mean that anti-insulin resistance bioactivity was unchanged or reduced and thus the index RGC is not suitable to evaluate anti-insulin resistance bioactivity.

Findings support that an evaluation index of glucose consumption ability (GCA = RGC/CV) that takes cell viability (CV) into account along with RGC is a more rational indicator than simply RGC for assessing anti-insulin resistance bioactivity of medicinal plant extracts. Among the eight traditional medicinal plants that demonstrated activities in increasing anti-insulin resistance bioactivity or enhancing glucose uptake, six were identified as effective according to the GCA indicator including *C. chinensis*, *M. alba*, *P. lobata*, *T. kirilowii*, *A. orientale* and *S. chinensis*. In addition, *A. membranaceus* and *C. chinensis* were identified as ineffective, while these species have previously been reported to show anti-diabetic capacity.

## Experimental Section

### General

Dulbecco’s modified eagle medium (DMEM) and fetal bovine serum (FBS) were purchased from Gibco (Shanghai, China). Glucose assay kits were purchased from Changchun Huili Biotech Co., Ltd (Changchun, China). Cell counting kit-8 (CCK-8) was provided by Dojindo Laboratorise (Shanghai). Dexamethasone (DXMS), insulin and DMSO were purchased from Sigma (Shanghai, China).

### Identification of Target Anti-diabetic Plants

Plants for the evaluation of anti-insulin resistance bioactivity were identified using an ethnobotanical approach based on the literature as well as primary research by this study’s authors. In addition, plants were selected on the basis of the criteria that they have not previously been evaluated for their anti-insulin resistance bioactivity in DXMS-induced IR HepG2 cells.

The literature review focused on identifying the most commonly used prescription anti-diabetic medicinal plants according to the frequency of usage recorded in six ancient Chinese medical manuscripts along with traditional medicinal plant articles published during 1980–2003 [17–20]. The six ancient Chinese medical manuscripts include: “Shen Nong Ben Cao Jing”, “Ming Yi Bie Lu”, “Bei Ji Qian Jin Yao Fang”, “Qian Jin Yi Fang”, “Dian Nan Ben Cao”, and “Ben Cao Gang Mu”. “Xiaoke” was used as a key word to select traditional medicinal plants in six ancient Chinese medical manuscripts. A total of nine plants were identified through literature reviews and were delineated as Group I plants (No. A1–A9 in Table 1).

**Fig. 1:**
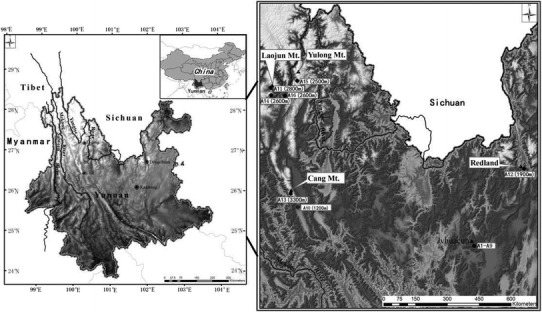
Map of ethnobotanical survey and medicinal plants material collection sites in Yunnan, China

The ethnobotanical survey involved plants mentioned in 2011–2013 during the author’s surveys in Lijiang, Dali, and Dongchuan of northern Yunnan Province of southwestern China (Fig. 1) with Han, Naxi, Bai, and Lisu socio-linguistic groups for the treatment of “xiaoke” and diabetic symptoms. Plants were identified through semi-structured interviews with traditional herb doctors that were over 50 years of age with an average age of 62. A total of ten key informants were interviewed including eight men and two women. A total of seven plants were identified through ethnobotanical survey and were delineated as Group II plants (No. A10–A16 in Table 1).

A total of 16 species from 14 plant families were selected for bioactivity screening and were divided into two groups (Table 1). Group I were purchased from Jv Hua Cun in Kunming, Yunnan Province of southwest China. The second group of focal plants were collected from ethnobotanical surveys in northern Yunnan Province. Voucher specimens of the 16 study species were deposited at the State Key Laboratory of Phytochemistry and Plant Resources Sustainability at the Kunming Institute of Botany of the Chinese Academy of Sciences (Kunming, China).

### Cell Line and Cell Culture

Human hepatoma cell line HepG2 was purchased from the Laboratory Animal Center of Sun Yat-Sen University (Guangzhou, China) and was maintained in DMEM containing 2.0 g/L of glucose and 10 % of FBS.

### Sample Preparation

Dried plant materials (500 g each) were pulverized and extracted with 4 L methanol at room temperature for 24 h. Each sample was extracted three times and resulting extracts were combined. Methanol extracts (MEs) were filtered and concentrated in vacuum. Extracts were dissolved in DMSO as stock solutions of 100 mg/mL and stored at −20 °C. For drug screening, the stock solution was thawed and diluted with cell growth medium to concentrations of 100, 50 and 25 μg/mL.

### Drug Treatment and Glucose Consumption Assay

Drug treatment proceeded by seeding 8 × 10^3^ HepG2 cells in 100 μL growth medium in 96-well plates. Plates were incubated for 24 h to allow cells to adhere to the well bottom. The medium was replaced with 100 μL fresh medium containing 100, 50 or 25 μg/mL of the medicinal plant extract or a blank without any extract. Drug treatment lasted for 48 h. To induce insulin resistance of cells, 1 μmol/L of DXMS was added to the medium with the medicinal plant extract [49]. After 48 h of drug treatment, 5 μL of medium was taken from each well for measurement of glucose concentration by the glucose assay kits. Glucose consumed in drug-treated wells was calculated and expressed as Relative Glucose Consumption (RGC) over that in drug free wells (insulin sensitive control, IS control). In this assay, RGC in drug free wells was set as 100 %.

### CV Assay

After removing 5 μL of medium from each well for the glucose consumption assay, 10 μL of CCK-8 was added per well in the 96-well plate and was further incubated at 37 °C for 1.5 h. Optical density (OD), which is positively correlated with the number of living cells, was read at 450 nm. Wells that were free of cells and that contained equivalent volumes of medium as the treated cells were set as blanks. Cell Viability (CV) in the IS control well was set as 100 %. CV in drug treated wells was calculated as CV (%) = 100 × (OD_Drug_ − OD_Blank_)/(OD_IS Control_ − OD_Blank_) where CV < 90 % indicates toxicity to cells [50].

### Evaluation of Glucose Consuming Ability of Cells

Anti-insulin resistance agents enhance glucose consumption and increase the Glucose Consuming Ability (GCA) of IR cells. Medicinal plant species used for treating diabetic symptoms were evaluated for their anti-insulin resistance bioactivity by employing DXMS-induced IR HepG2 cells as an IR cell model and Glucose Consuming Ability (GCA) as an evaluation indicator.

GCA was calculated as GCA = RGC/CV. For the IS control which has RGC and CV values of 100 %, GCA_IS_ = 1. For DXMS-induced IR HepG2 cells (IR control) which has reduced glucose uptake, GCA_IR_ < 1. For IR cells treated with a medicinal plant extract, a GCA_IR(Extract)_ > GCA_IR_ indicates that the extract enhanced glucose uptake and increased anti-insulin resistance bioactivity of IR cells. A GCA_IR(Extract)_ < GCA_IR_ indicates that the extract did not enhance glucose uptake. In this study, GCA_IR(Extract)_ ≥ 1 suggests remarkable bioactivity of the extract.

## Conclusion

This study used interdisciplinary research methods that integrated ethnobotany, phytochemistry, and pharmacology towards exploring traditional medicinal plants for drug discovery. Findings validate traditional usage of medicinal plants through scientific explanation of their efficacy for the prevention and treatment of diabetic symptoms and provide a promising knowledge base for drug discovery to mitigate the global diabetes epidemic. Further studies should incorporate traditional as well as novel experimental preparation protocols of plant remedies for drug discovery to evaluate both efficacy and safety. In addition, future research should adopt an activity-guided fractionation approach to drug discovery by assessing different fractions and chemical constituents of the medicinal plants examined in this study along with in vivo screening. The evaluation model used here, which combines cell viability of DXMS-induced IR HepG2 cells and GCA, is a reasonable method for evaluating anti-insulin resistance bioactivity and should be utilized widely as a screening technique for drug discovery from plant resources. Glucose consuming ability of cell (GCA) is only one of useful evaluation indicators in screening of anti-insulin resistance bioactivity and different evaluation models need to be tested and verified. The integration of traditional healing systems with scientific research can contribute to the management of global health epidemics including DM while providing economic incentives to conserve plant resources of traditional systems and their associated cultural practices and knowledge base.

## Abbreviations

DM: Diabetes mellitus
IR: Insulin resistant
IS: Insulin sensitive
DXMS: Dexamethasone
RGC: Relative glucose consumption
CV: Cell viability
GCA: Glucose consuming ability
MEs: Methanol extracts
